# microRNA-26a suppresses recruitment of macrophages by down-regulating macrophage colony-stimulating factor expression through the PI3K/Akt pathway in hepatocellular carcinoma

**DOI:** 10.1186/s13045-015-0150-4

**Published:** 2015-05-29

**Authors:** Zong-Tao Chai, Xiao-Dong Zhu, Jian-Yang Ao, Wen-Quan Wang, Dong-Mei Gao, Jian Kong, Ning Zhang, Yuan-Yuan Zhang, Bo-Gen Ye, De-Ning Ma, Hao Cai, Hui-Chuan Sun

**Affiliations:** Liver Cancer Institute, Zhongshan Hospital, Fudan University, 136 Yi Xue Yuan Road, Shanghai, 200032 People’s Republic of China; Key Laboratory of Carcinogenesis and Cancer Invasion, Ministry of Education, Fudan University, 136 Yi Xue Yuan Road, Shanghai, 200032 People’s Republic of China; Department of Pancreatic and Hepatobiliary Surgery, Fudan University Shanghai Cancer Center, Shanghai, People’s Republic of China; Department of Oncology, Shanghai Medical College, Fudan University, Shanghai, People’s Republic of China; Pancreatic Cancer Institute, Fudan University, Shanghai, People’s Republic of China; Department of Hepatobiliary Surgery, Beijing Chaoyang Hospital, Capital Medical University, Beijing, People’s Republic of China

**Keywords:** microRNA-26a, Hepatocellular carcinoma, M-CSF, Macrophages

## Abstract

**Background:**

microRNAs (miRNAs) have been reported to modulate macrophage colony-stimulating factor (M-CSF) and macrophages. The aim of this study was to find whether miR-26a can suppress M-CSF expression and the recruitment of macrophages.

**Methods:**

Hepatocellular carcinoma (HCC) cell lines with decreased or increased expression of miR-26a were established in a previous study. M-CSF expression by tumor cells was measured by enzyme-linked immunosorbent assay, and cell migration assays were used to explore the effect of HCC cell lines on macrophage recruitment in vitro. Real-time PCR measured a panel of mRNAs expressed by macrophages. Xenograft models were used to observe tumor growth. Immunohistochemistry was conducted to study the relation between miR-26a expression and M-CSF expression and macrophage recruitment in patients with HCC.

**Results:**

Ectopic expression of miR-26a reduced expression of M-CSF. The conditioned medium (CM) from HepG2 cells that overexpressed miR-26a reduced the migration ability of THP-1 cells stimulated by phorbol myristate acetate (PMA) increased expression of interleukin (IL)-12b or IL-23 mRNA and decreased expression of chemokine (C-C motif) ligand (CCL)22, CCL17, and IL-10 mRNA, in comparison to the medium from the parental HepG2 cells. These effects could be interrupted by the PI3K/Akt pathway inhibitor LY294002. Ectopic expression of miR-26a in HCC cells suppressed tumor growth, M-CSF expression, and infiltration of macrophages in tumors. Similar results were also found when using HCCLM3 cells. Furthermore, the expression of miR-26a was inversely correlated with M-CSF expression and macrophage infiltration in tumor tissues from patients with HCC.

**Conclusions:**

miR-26a expression reduced M-CSF expression and recruitment of macrophages in HCC.

## Background

Hepatocellular carcinoma (HCC) is the sixth most common cancer and third leading cause of cancer-related death worldwide [1]. Treatment of HCC has changed greatly in recent years, but hepatic resection and transplantation are still considered the main curative therapy, and a high postoperative recurrence rate is a major impediment to prolonging patient survival [2].

Crosstalk between tumor cells and their microenvironment underlies the pathogenesis of HCC [3]. Transplantation of normal hepatocytes into a neoplastic-prone liver microenvironment delays the growth of hepatic nodules and the emergence of HCC [4]. A unique immune/inflammation response signature is associated with HCC intrahepatic metastasis, and inflammatory cytokines can predict poor survival of patients with HCC [5, 6]. These findings suggest that the immune/inflammation microenvironment may foster the development of HCC. Tumor-associated macrophages (TAMs) are key components of the cancer microenvironment [7, 8]. TAMs originate from circulating monocyte precursors that are recruited to the tumor by tumor-derived signals, including chemokine (C-C motif) ligand 2 (CCL2) and macrophage colony-stimulating factor (M-CSF). In response to distinct microenvironment signals, macrophages can exert either anti- or pro-tumor activities [9], classified as M1 (or classical) or M2 (or alternatively activated), respectively [10, 11]. In HCC, most macrophages in the peritumor region exhibit an M2 phenotype, which is probably determined by the tumor cell [12, 13]. These macrophages facilitate tumor growth, metastasis, and angiogenesis and are associated with poor patient survival [13–16]. In many types of tumors, M-CSF is an essential regulator or recruiter of macrophages and is mainly produced by tumor cells [17, 18]. After binding to colony-stimulating factor 1 receptor (CSF-1R) on macrophages, M-CSF can activate macrophages, promoting secretion of growth factors that are essential for the pre-metastatic niche and tumor growth or metastasis [19–21]. Some studies have also found high expression of M-CSF and its receptor in peritumoral liver tissue, which is associated with poor survival of patients with HCC after curative resection [6, 22].

microRNAs (miRNAs) are a class of 22-nucleotide noncoding RNAs. The aberrant expression of specific miRNAs is directly involved in tumorigenesis, including growth, apoptosis, angiogenesis, and metastasis [23–27] and affects the biology of cellular components belonging to the tumor microenvironment, including endothelial cells, pericytes, fibroblasts, and immune cells [28–31]. In HCC, miRNAs have been reported to regulate the biology of endothelial cells and Treg cells [32, 33]. It has also been reported that miR-214 modulates macrophage polarization in HCC [34]. However, no study has addressed whether miRNAs regulate M-CSF expression in tumor cells and the recruitment of macrophages in HCC.

Deregulation of miR-26a may function as either a tumor suppressor or promoter. miR-26a is down-regulated in breast cancer, anaplastic carcinomas, and oral squamous cell carcinoma [35–38] but up-regulated in cholangiocarcinoma and glioma [39–41]. In HCC, a reduced level of miR-26a is associated with poor overall survival of patients with HCC [42]. Patients with hepatitis B virus-related HCC had a lower level of miR-26a in blood compared with patients with chronic hepatitis B [43]. In addition, miR-26a can inhibit cancer cell proliferation and protect against disease progression without toxicity in an HCC mouse model [44–46]. The underlying mechanisms might be that miR-26a can inhibit tumor growth, metastasis, and angiogenesis in HCC [27, 44, 47, 48]. However, no study has addressed the role of miR-26a in TAMs.

In the present study, we investigated whether miR-26a regulates recruitment and function of macrophages in HCC and the underlying mechanisms of miR-26a.

## Results

### miR-26a suppressed the attraction of macrophages by inhibiting M-CSF secretion

In a previous study [27], we found miR-26a suppressed the activation of the PI3K/Akt signal pathway. Because M-CSF is an important cytokine for macrophage recruitment and can be modulated by the PI3K/Akt signal pathway [49], we investigated whether miR-26a can regulate the expression of M-CSF and the attraction of macrophages in HCC.

**Fig. 1:**
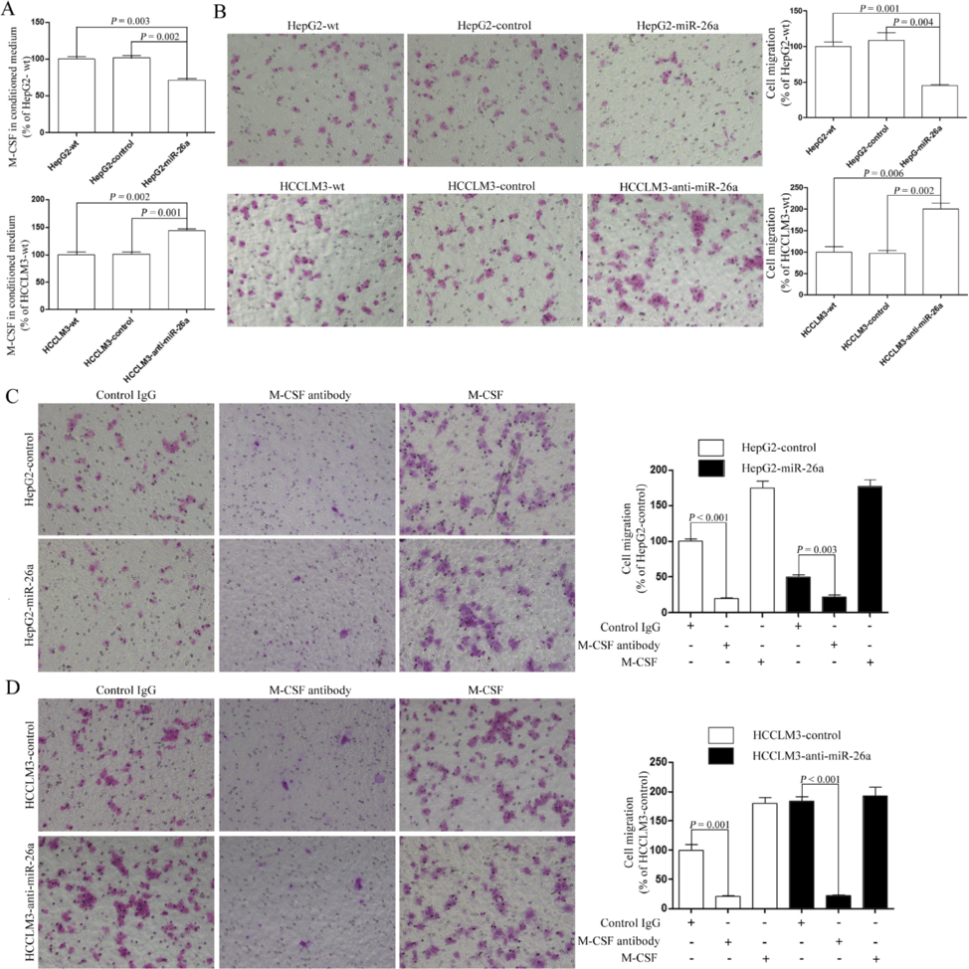
miR-26a suppressed the motility of macrophages by inhibiting M-CSF secretion. **a** After the modifications of miR-26a in HCC cell lines, the level of M-CSF in the CM of HCC cells was detected by ELISA. **b** THP-1 cells stimulated by PMA were treated with CM from all HCC cells, and their migration ability was assessed. **c, d** THP-1 cells stimulated by PMA were treated with CM from HCC cells with addition of M-CSF neutralizing antibody or control IgG, and their migration was assessed

We first collected conditioned medium (CM) from HCC cell lines established in our previous study that stably expressed miR-26a at different levels. These cells lines included HepG2-wt (wild type of HepG2), HepG2-control (HepG2 transfected with the negative control of precursor miRNA), and HepG2-miR-26a (HepG2 transfected with pre-miR-26a) and HCCLM3-wt (wild type of HCCLM3), HCCLM3-control (HCCLM3 transfected with the negative control of anti-miRNA-locked nucleic acids [LNAs]), and HCCLM3-anti-miR-26a (HCCLM3 transfected with anti-miRNA-LNAs against miR-26a). Then, the protein level of M-CSF was detected by enzyme-linked immunosorbent assay (ELISA). We found that secretion of M-CSF was significantly decreased in the CM of HepG2-miR-26a but significantly increased in HCCLM3-anti-miR-26a (Fig. 1a).

To explore whether miR-26a affects motility of macrophages, a migration assay was conducted using THP-1 cells stimulated by PMA. We found that the migration of THP-1 cells was inhibited by the CM of HepG2-miR-26a cells and induced by the CM from HCCLM3-anti-miR-26a cells (Fig. 1b) in comparison to CM from the control cells.

To detect whether the inhibitory effect of miR-26a on motility of macrophages was mediated by M-CSF, we added M-CSF-neutralizing antibody and human M-CSF protein into the CM we collected. The results showed that THP-1 cell migration was suppressed by the CM of HepG2 cells treated with M-CSF-neutralizing antibody and induced by the CM of HepG2 cells treated with the extrinsic M-CSF protein. Furthermore, the difference in migration ability between HepG2-control and HepG2-miR-26a cells was eliminated (Fig. 1c). Similar results were also found in HCCLM3 cells (Fig. 1d).

### miR-26a inhibited the expression of M-CSF by regulating the PI3K/Akt pathway

**Fig. 2:**
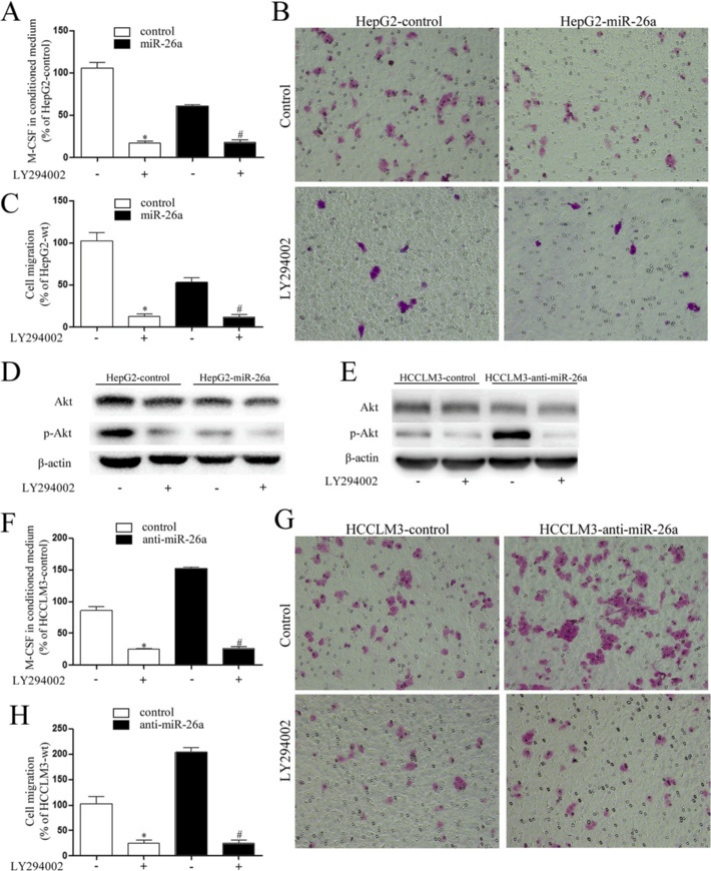
The inhibiting effect of miR-26a on macrophages mainly through regulation of the PI3K/Akt pathway. LY294002 was used to treat HCC cells, and the CM was collected. **a, f** The expression levels of M-CSF in the CM of all HCC cells were assessed by ELISA. **b, c, g, h** The effect of CM on migration of THP-1 cells stimulated by PMA was assessed. **d, e** The expression of Akt and p-Akt in HCC cells were examined by Western blot

To explore whether miR-26a affects M-CSF expression through the PI3K/Akt pathway, the PI3K/Akt pathway inhibitor LY294002 was used. The results showed that expression of M-CSF in HepG2 cells was inhibited by LY294002 (Fig. 2a), and p-Akt was down-regulated, but the total Akt expression was not affected (Fig. 2d). Migration of THP-1 cells was inhibited (Fig. 2b, c). LY294002 treatment also eliminated the difference in M-CSF secretion and migration of THP-1 cells among HepG2-wt, HepG2-control, and HepG2-miR-26a cells. Similar results were also found in HCCLM3 cells (Fig. 2e–h).

### miR-26a affected polarization of macrophages

**Fig. 3:**
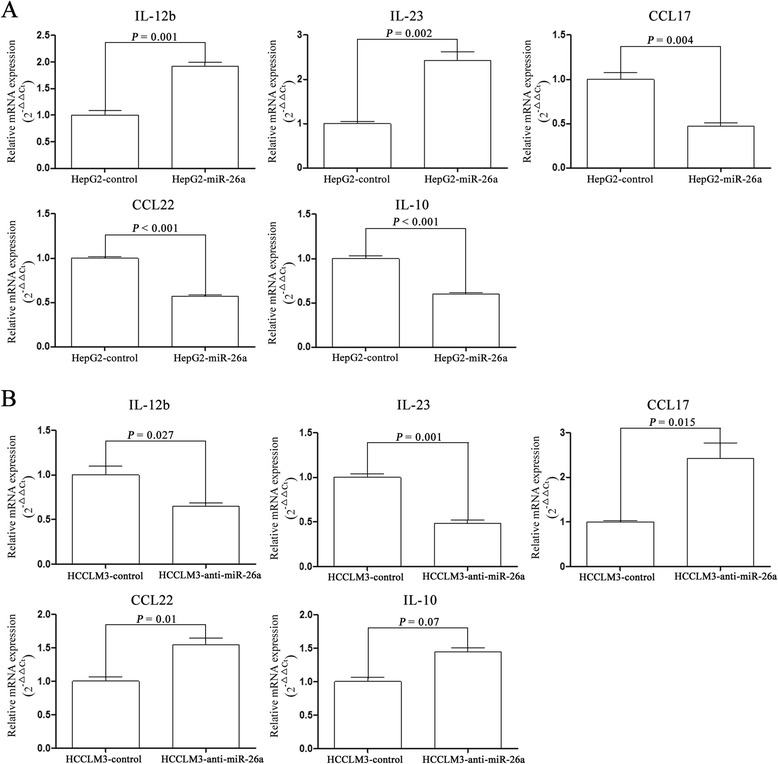
The regulatory effect of miR-26a on a panel of mRNA expressed by macrophages. THP-1 cells stimulated by PMA were treated with CM from HCC cells. The mRNA expression of IL-12b, IL-23, IL-10, CCL17, and CCL22 in the THP-1 cells treated with CM from HepG2-miR-26a and HepG2-control cells (**a**) or HCCLM3-anti-miR-26a and HCCLM3-control cells (**b**) were detected by RT-PCR

Because M1 macrophages produce higher levels of interleukin (IL)-12b and IL-23 and lower levels of IL-10, CCL17, and CCL22 compared with M2 macrophages [50, 51], we examined mRNA expression of these cytokines in the THP-1 cells stimulated by PMA and treated with the CM of HCC cells to study whether miR-26a affects polarization of macrophages. The results showed that the THP-1 cells treated with CM of HepG2-miR-26a cells expressed more IL-12b and IL-23 mRNA and less CCL22, CCL17, and IL-10 mRNA in comparison with THP-1 cells treated with the CM of HepG2-control cells (Fig. 3a). In contrast, the THP-1 cells treated with CM of HCCLM3-anti-miR-26a cells had lower IL-12b and IL-23 mRNA expression but higher CCL22, CCL17, and IL-10 mRNA expression.

### miR-26a inhibited tumor growth and recruitment of macrophages in HCC xenograft models

**Fig 4:**
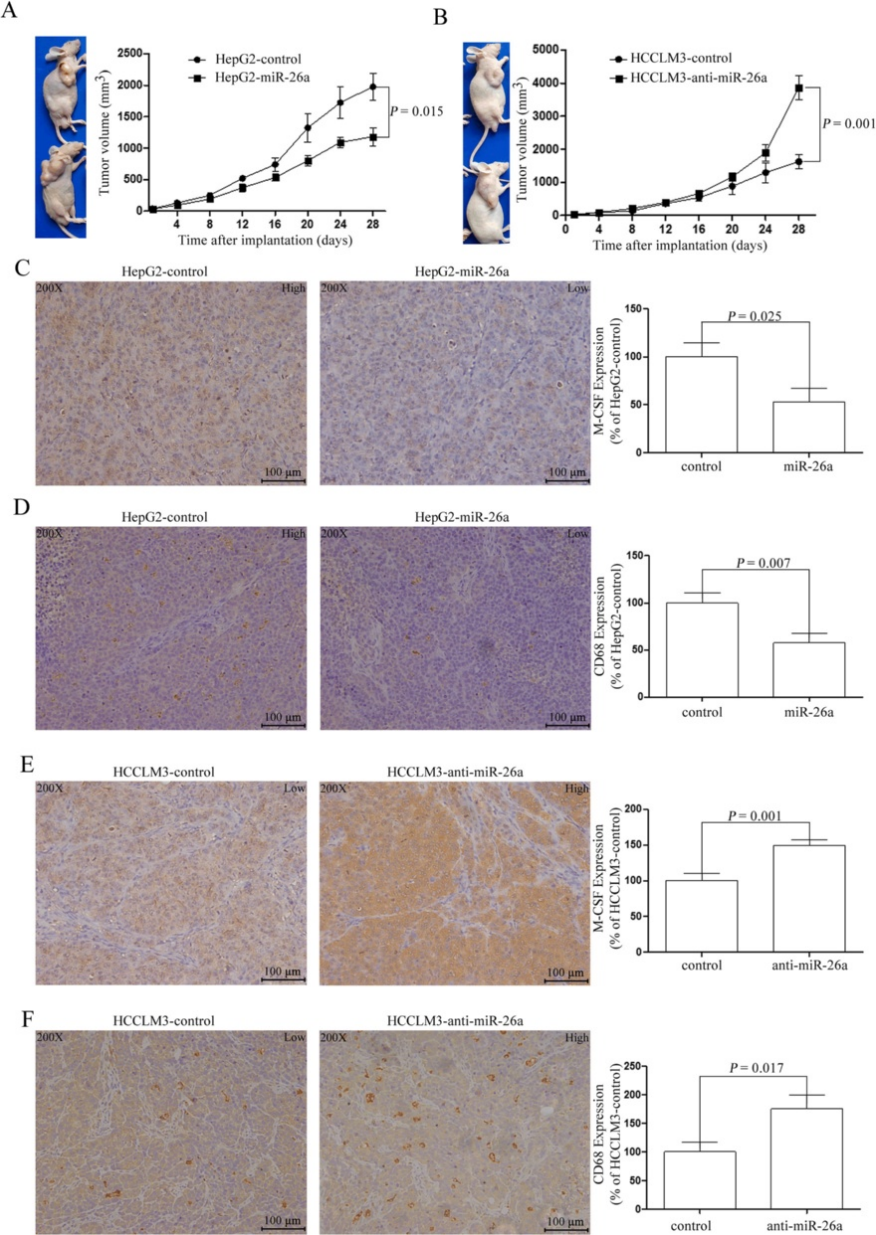
The role of miR-26a on M-CSF expression and recruitment of macrophages in vivo. HCC cells were injected into the right flanks of nude mice. **a, b** The tumor growth curves of subcutaneous tumors were assessed. **c, d** The M-CSF expression and CD68 expression in the tumors of HepG2 cells were assessed by immunohistochemistry. **e, f** The M-CSF expression and CD68 expression in the tumors of HCCLM3 cells were assessed by immunohistochemistry. Representative images are shown (×200)

To evaluate the role of miR-26a on recruitment of macrophages, we established HepG2 and HCCLM3 xenograft models. Similar to our previous findings [27], HepG2-miR-26a tumors had a decreased tumor volume, while HCCLM3-anti-miR-26a tumors had an increased volume compared with their parental cell lines, respectively (Fig. 4a, b). Further investigation of the M-CSF expression and infiltrating macrophages revealed that HepG2-miR-26a tumors had lower M-CSF expression and a decreased number of infiltrating macrophages compared with HepG2-control tumors (Fig. 4c, d). HCCLM3-anti-miR-26a tumors had higher M-CSF expression and fewer macrophages compared with HCCLM3-control tumors (Fig. 4e, f).

### miR-26a expression was inversely correlated with M-CSF expression and infiltrating macrophages in tumors from patients with HCC

**Fig. 5:**
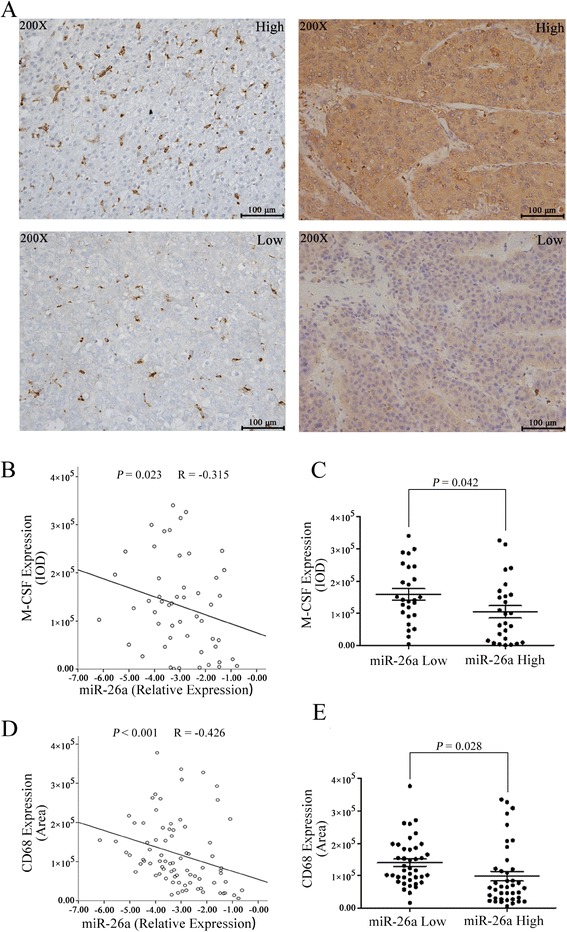
The relationship between miR-26a and the expression M-CSF and the density of macrophages in HCC tissue. **a** The HCC tissue microarrays were used to analyze M-CSF expression and density of macrophages through immunohistochemistry. Representative images are shown (×200). **b, c** The relationship between M-CSF expression and miR-26a expression was analyzed. **d, e** The relationship between the density of macrophages and miR-26a expression was analyzed. IOD integrated optical density

To examine whether miR-26a expression was correlated with M-CSF expression and the density of macrophages, we examined M-CSF expression in 52 patients with HCC and CD68 expression in 80 patients with HCC. miR-26a expression data were obtained in our previous study [42]. The representative images of M-CSF and CD68 immunostaining are shown in Fig. 5a. The statistical results for M-CSF expression showed that miR-26a expression was inversely correlated with the M-CSF expression (Fig. 5b). After dividing the patients into two equal groups, using the median value of miR-26a expression as the cutoff point, we found that patients with higher miR-26a expression had lower M-CSF expression compared with patients with lower miR-26a expression (Fig. 5c). We found similar results in the analysis of the relation between miR-26a and CD68 expression (Fig. 5d, e).

## Discussion

miR-26a has been reported to be able to reduce proliferation, metastasis, and angiogenesis in HCC [27, 44, 47, 48]. We further demonstrated that miR-26a can regulate the recruitment of macrophages. Some studies have reported miRNAs associated with macrophage infiltration, apoptosis, and phenotype. For example, let-7d suppresses macrophage infiltration by targeting COL3A1 and CCL7 [52], miR-142-5p can regulate apoptosis of macrophages by targeting TGF-β2 [53], and miR-155 can modulate macrophage polarization by targeting C/EBP-β [54]. Here, we first demonstrated that miR-26a can suppress macrophage recruitment by regulating M-CSF expression in HCC.

In our previous study, we found that miR-26a expression was lower in HCC tumor samples than in paired noncancerous liver tissue, and patients with lower miR-26a expression had shorter overall survival compared with patients with higher miR-26a expression [42]. The underlying mechanism related to miR-26a may be suppression of HCC cell proliferation and metastasis by regulation of the IL-6-stat3 signaling pathway and inhibition of angiogenesis in HCC by down-regulating Akt/VEGF signaling [27, 47, 48]. In addition, Kota et al. [44] reported that miR-26a can inhibit cancer cell proliferation in an HCC mouse model by targeting cyclin D2 and E2. However, the underlying mechanisms for how miR-26a affects the survival of patients with HCC are still not fully understood. The present study was designed to show a new role of miR-26a in the recruitment of macrophages in HCC.

M-CSF is one of the major cytokines controlling the proliferation, differentiation, and functional regulation of macrophage [55]. The expression level of M-CSF has been found to be associated with higher histological tumor grading, more frequent metastases, and poor prognosis in many cancer types, including papillary renal cell carcinoma, serous and mucinous ovarian epithelial tumors, endometrioid carcinomas, breast cancer, and especially HCC [51, 56–59]. Our previous study showed that high expression of M-CSF in peritumoral liver tissue is associated with poor survival after curative resection of HCC [6, 22]. Various miRNAs have been reported to be involved in the regulation of M-CSF expression. Mandal et al. [49] found miR-21a can induce M-CSF expression by regulating the PI3K/Akt signal pathway. Cimino et al. [60] reported M-CSF is a direct target of miR-148b. Wang et al. [61] identified M-CSF as a target gene of miR-214, and Zhang et al. [62] reported M-CSF as a target of miR-128. The present study found miR-26a regulates M-CSF expression and recruitment of macrophages. In addition, M-CSF also plays an important role in the polarization of macrophages. It can stimulate macrophages and induce them to exhibit an anti-inflammatory M2-type of activation, which produces higher IL-12/23 but lower CCL17, CCL22, and IL-10 [50, 51, 63, 64]. The present study also found miR-26a can induce a pro-inflammatory M1-type of activation for macrophages and may affect the overall survival of patients with HCC partly through reducing macrophage recruitment and down-regulating M-CSF expression.

This study has some potential limitations. We found miR-26a can inhibit macrophage recruitment by down-regulating M-CSF expression, but recruitment might be influenced by many factors. Our preliminary study showed miR-26a expression did not affect expression of CCL2 and GM-CSF (data not shown), which are two major cytokines regulating recruitment of macrophages [51, 65].

M-CSF inhibitor has been reported to impede macrophage recruitment in malignancy [66]. For HCC, zoledronic acid, a bisphosphonate with a macrophage-modulating effect, can inhibit growth of HCC cells and delay disease progression of bone metastases [67, 68]. Our previous study reported that depletion of macrophages using zoledronic acid can enhance the effect of sorafenib in HCC as well [69]. With all data considered together, M-CSF and macrophage might be potential therapeutic targets for HCC. Our data provide evidence that miR-26a may suppress the recruitment of macrophages by down-regulating M-CSF expression. The data also suggest that miR-26a may be a marker for the grade of malignancy in HCC as well as a potential therapeutic target in patients with HCC.

## Methods

### Cell lines

The HCC cells which were altered expression levels of miR-26a by using recombinant lentivirus vector (Genechem, Shanghai, China) were established in our previous study. Human monocyte cell line THP-1 were obtained from Shanghai Institute of Cell Biology (Shanghai, China). The HCC cells were cultured in Dulbecco’s modified Eagle’s medium (DMEM, Invitrogen, Carlsbad, CA) containing 10 % fetal bovine serum (FBS), and THP-1 cells were cultured in RPMI 1640 (Invitrogen, Carlsbad, CA) supplemented with 10 % FBS. All cells were maintained in a humidified incubator at 37 °C with an atmosphere of 5 % CO_2_.

### Collection of the CM

HCC cells were treated with LY294002 (20 μM, Beyotime, Jiangsu, China) or vehicle for 12 h, then incubated in DMEM with 0.1 % BSA for 24 h. The CM was centrifuged for 20 min at 3000 rpm, and the resultant pellet was stored at −80 °C. In the M-CSF blocking assay experiments, neutralizing antibody against M-CSF (R&D Systems, Minneapolis, MN) and control IgG were added to CM 30 min before further experiments.

### Quantification of M-CSF in the CM

ELISA (R&D Systems) was used to measure M-CSF concentrations in the CM. The total protein concentration was measured by bicinchoninic acid (BCA) assay, and the M-CSF concentration was normalized according to the total cellular protein.

### Cell migration assay

Chambers (Corning, Tewksbury, MA) with 8.0-μm polycarbonate filter inserted in 24-well plates were used in the quantitative cell migration assays as described before [27]. THP-1 cells stimulated by PMA (1 × 10^5^ cells/well) were added in the upper chamber, and the lower chamber was filled with the previously collected CM. The 24-well plates filled with cells were placed in a thermostatic incubator at 37 °C for 16 h. Thereafter, the migrated cells were fixed with methanol, stained with crystal violet, and photographed under an inverted microscope. The areas of stained cells from three random fields at × 200 magnification were assessed by using Image-Pro Plus software (Media Cybernetics Inc, Bethesda, MD).

### Western blot assay

As described in our previous study [27], cells were lysed in buffer (150 mM NaCl, 50-mM Tris-HCl, pH 8.0, 0.1 % SDS, 1 % Triton X-100) containing protease and phosphatase inhibitors. Fifty micrograms of whole cell extracts were subjected to SDS-PAGE gel and transferred to nitrocellulose membranes. The membranes were blocked with 5 % nonfat milk for 2 h and then incubated with respective primary antibody overnight at 4 °C, followed by incubation with the appropriate HRP-conjugated secondary antibody for 2 h at room temperature. Blots were visualized with an ECL detection kit (Pierce, IL) and analyzed using Quantity One 1-D Analysis Software (Bio-Rad, San Francisco, CA).

### Real-time PCR assay

Equal amounts of THP-1 cells stimulated by PMA were treated with the previously collected CM. After 48 h, total RNA was extracted using TRIzol Reagent (Sigma, St. Louis, MO). As described before [69], reverse transcription reactions and quantitative real-time PCR were performed using RT-PCR kit (TaKaRa, Shiga, Japan). The primers used for the amplification of human genes are listed as follows: IL-12 (5′-TGCCCATTGAGGTCATGGTG-3′ [forward]; 5′-CTTGGGTGGGTCAGGTTTGA-3′ [reverse]), IL-23 (5′-CAGAGAGAATCAGGCTCAAAGC-3′ [forward]; 5′-AGCAACAGCAGCATTACAGC-3′ [reverse]), CCL17 (5′-CTGGGACCTCCACCGTT-3′ [forward]; 5′-CTCACTGTGGCTCTTCTTCGT-3′ [reverse]), CCL22 (5′-AAACTAATGTCCCTCCCCTCTC-3′ [forward]; 5′-TTTGGGGCTTCACATTGACC-3′ [reverse]), IL-10 (5′-TGCCTAACATGCTTCGAGATC-3′ [forward]; 5′-CCAGGTAACCCTTAAAGTCCTC-3′ [reverse]), and ACTB (5′-ACTGGGACGACATGGAGAAAATC-3′ [forward]; 5′-CTCGCGGTTGGCCTTGG-3′ [reverse]).

### Xenograft model of human HCC in nude mice

As described in our previous study [27], male BALB/c nude mice (5 weeks old) were purchased from the Shanghai Institute of Materia Medica, Chinese Academy of Science, and housed under specific pathogen-free conditions. The experimental protocol was approved by the Shanghai Medical Experimental Animal Care Commission. Twenty mice were randomized into four groups, and various cancer cells (6 × 10^6^ cells) in 200 μl of normal saline were implanted by subcutaneous injection to obtain subcutaneous tumors. Tumor dimensions were measured by vernier caliper every 4 days, and the mice were killed after 4 weeks. After final measurement, the tumors were placed in 4 % paraformaldehyde solution. The tumor volume was calculated according to the formula: tumor volume = (largest diameter × perpendicular height^2^)/2.

### Immunohistochemical assay

Paraffin-embedded tumor tissues from animal Immunohistochemistry was performed on the sections or tissue arrays containing tumor tissues from patients with HCC who had antibodies against M-CSF (1:100; Abcam, Cambridge, MA) or CD68 (1:100; Abcam, Cambridge, MA). The integrated optical density (for M-CSF) or area (for CD68) of positive staining/total area was quantified by Image-Pro Plus software [27]. For the reading of each antibody staining, a uniform setting for all the slides was applied.

### Patients and follow-up

HCC specimens used in the immunohistochemical assay were obtained from patients who received radical resection between 1999 and 2003 at the Liver Cancer Institute and Zhongshan Hospital (Fudan University, Shanghai, China); these specimens have been described previously [27, 42]. None of the patients received any preoperative anticancer treatment. The research was approved by the research ethics committee of Zhongshan Hospital. A total of 80 cases were used to examine CD68 expression, and 52 cases were used to examine CD68 expression. The miR-26a expression data were collected in our previous study. All the patients were followed up until 2011 with a median observation time of 60.8 months. This study was approved by the Zhongshan Hospital Research Ethics Committee. All patients provided their written informed consent to participate in this study.

#### Statistical analysis

Data were analyzed using SPSS 18.0 (SPSS, Inc.). Quantitative variables were analyzed using the unpaired two-tailed Student’s *t* test and Spearman correlation test. *P* < 0.05 was considered statistically significant.

## Abbreviations

CCL17: Chemokine (C-C motif) ligand 17
CCL22: Chemokine (C-C motif) ligand 22
HCC: Hepatocellular carcinoma
IL-10: Interleukin-10
IL-12: Interleukin-12
IL-23: Interleukin-23
M-CSF: Macrophage colony-stimulating factor
miR-26a: microRNA-26a
TAMs: Tumor-associated macrophages
